# Mask Ventilation Failure During Induction of General Anesthesia in an Infant With Osteogenesis Imperfecta Type II

**DOI:** 10.7759/cureus.68059

**Published:** 2024-08-28

**Authors:** Tatsuya Abe, Yutaka Seino, Hidekazu Imai

**Affiliations:** 1 Department of Anesthesiology, Niigata University Medical and Dental Hospital, Niigata, JPN

**Keywords:** infant, ventilation failure, mask ventilation difficulty, difficult airway, osteogenesis imperfecta

## Abstract

Osteogenesis imperfecta (OI) is a congenital disease characterized by fractures and progressive bone deformities due to systemic bone fragility. A 12-month-old male infant diagnosed with OI type II, the most severe type, was scheduled for a tracheostomy. The patient presented with thoracic hypoplasia, which was treated with a high-flow nasal cannula, and a large skull owing to hydrocephalus with the head fixed in a left anteversion position. We encountered difficulties in mask ventilation during the rapid induction of general anesthesia. Oxygen saturation dropped temporarily, but the patient’s condition stabilized after intubation. The tracheostomy was performed as scheduled and was completed without any complications. Difficulty in mask ventilation with low thoracic compliance due to thoracic hypoplasia, combined with air-induced gastric dilatation and upper airway obstruction, may have contributed to the ventilation failure. Most OI type II patients have large skulls owing to hydrocephalus and thoracic hypoplasia. Since no iatrogenic fractures related to airway management were observed in this or past cases, bone fragility may not be concerning to the extent that airway management becomes compromised. If patients present with poor oxygenation due to thoracic hypoplasia, the possibility of difficulty with mask ventilation due to low compliance should be considered in airway management.

## Introduction

Osteogenesis imperfecta (OI) is a congenital disease caused by genetic mutations in type I collagen, a major component of connective tissue, resulting in bone fractures and progressive bone deformities due to systemic bone fragility. Anesthetic considerations include iatrogenic fractures, respiratory compromise due to thoracic deformities and hypoplasia, elevated body temperature during general anesthesia, and bleeding tendencies [1]. There are several types of OI, but very few perioperative case reports have been published for type II, which is the most severe form of the disease. OI type II has an 80% mortality rate in seven days, and survival beyond a year is very rare. It usually requires intensive support, including continuous assisted ventilation [2]. Therefore, tracheostomy is not uncommon, and general anesthesia is required.

In this case, we experienced mask ventilation difficulty during general anesthesia induction in an infant with OI type II. The details of the case and the precautions for general anesthesia are discussed. Written informed consent for the publication of this report was obtained from the patient’s parents.

## Case presentation

**Figure 1:**
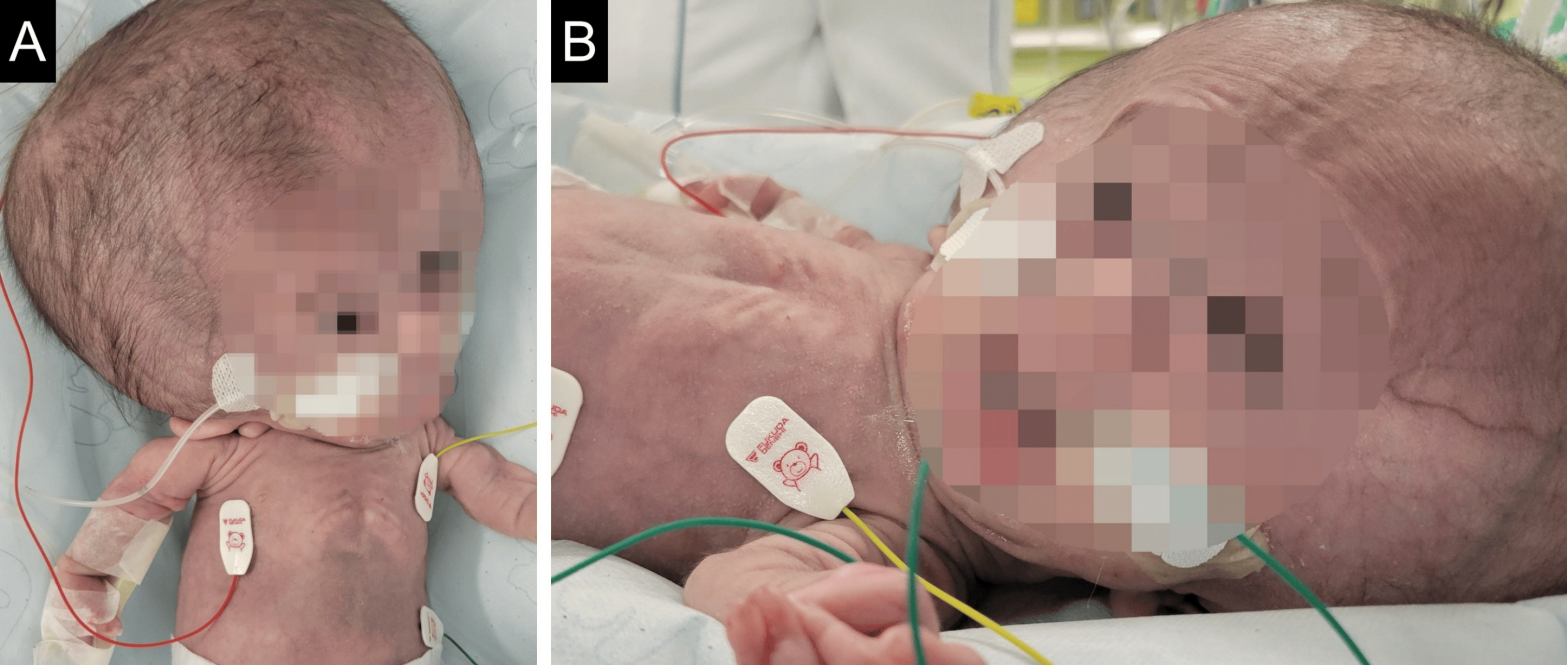
The patient's appearance The occipital shape is deformed such that the head is fixed in the left anteversion position. The airway is corrected by inserting a pillow under the shoulder and head.

A 12-month-old male infant diagnosed with OI type II (weight, 3116 g; height, 43 cm) was scheduled for a tracheostomy. The patient presented with thoracic hypoplasia and retractive breathing and was treated with a high-flow nasal cannula at gas flow of 8 L/minute and fraction of inspired oxygen (FiO_2_) of 0.35-0.4. The patient’s vital signs were a heart rate of 150-190 beats/minute, respiratory rate of 40-70 breaths/minute, and saturation of percutaneous oxygen (SpO_2_) of 93-98%. Blood pressure was not measured because of the risk of fracture due to manchette pressurization. The skull was large owing to hydrocephalus, and the occipital shape was deformed, so the head was fixed in a left anteversion position (Figure 1).

**Figure 2:**
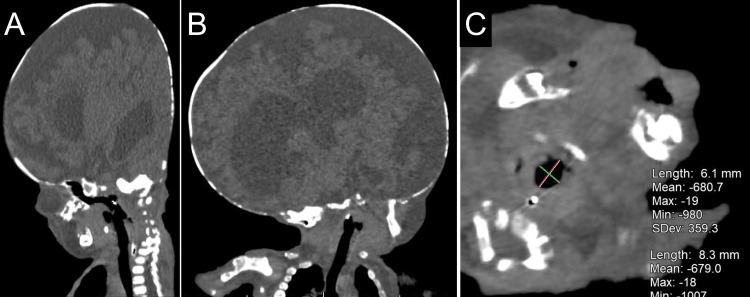
Computed tomography findings of the patient (A, B) No airway stenosis is observed. (C) The diameter of the narrowest part of the trachea is 6.1 x 8.3 mm, which is large relative to the body size.

Nevertheless, the pediatrician and the otolaryngologist successfully positioned the patient in a retroflexed position for the tracheostomy with gentle manipulation. Preoperative computed tomography (CT) showed no indications of airway narrowing (Figure 2). As the patient could be placed in the retroflexed position without difficulties and CT findings showed no issues, we considered that airway management could be performed as usual. The use of inhaled anesthetics was not included in the anesthesia plan because OI is associated with malignant hyperthermia [1].

General anesthesia was induced with atropine 0.05 mg, midazolam 0.5 mg, fentanyl 20 µg, and rocuronium 5 mg, following which the heart rate was 206 beats/minute and SpO_2_ was 100%. When mask ventilation was performed using the two-person method, capnography waveforms were almost undetectable. However, thoracic elevation was confirmed, and oxygenation was maintained. Although we attempted to lift the epiglottis using a Macintosh laryngoscope with a curved blade size one, the glottis was not visible. Mask ventilation was performed again, but the thorax was no longer elevated. We inserted an oral airway, corrected the mask fit, and suctioned the gastric contents from the gastric tube to exclude gastric dilatation caused by air delivery; however, no improvement was observed. The oxygen saturation dropped rapidly, becoming unmeasurable, and the patient became bradycardic with a heart rate of 61 beats/minute. A senior anesthesiologist successfully performed tracheal intubation using the same Macintosh laryngoscope and inserted a cuffed endotracheal tube with an inner diameter of 4.0 mm. An immediate increase in oxygen saturation and improvement in bradycardia were observed with ventilation. The ventilator settings were pressure-controlled with peak inspiratory pressure of 25-28 cmH_2_O, positive end-expiratory pressure of 4 cmH_2_O, and respiratory rate of 40 breaths/minute. The tidal volume was 20-25 mL, and low thoracic compliance was observed. The tracheostomy was performed as scheduled and completed without any complications. Spontaneous ventilation gradually stabilized after the patient returned to the neonatal intensive care unit. The patient was moving all extremities, and no perioperative iatrogenic fractures occurred, as far as we could confirm.

## Discussion

The patient had difficulty with mask ventilation. Low thoracic compliance due to thoracic hypoplasia combined with air-induced gastric dilatation and upper airway obstruction factors, such as tongue base obstruction, may have contributed to the ventilation failure.

Previous reports on airway problems in OI did not include type II [1,3,4]. No serious events occurred, such as mask ventilation difficulties or the need for surgical airway management, and OI itself was not a risk factor in these cases. A report on general anesthesia in a patient with OI type II described an event in which the glottis became edematous and mask ventilation became impossible because of repeated intubation maneuvers [5]. The case presented by To et al. [5] is similar to ours, with thoracic hypoplasia requiring ventilator support.

No objective assessments, such as pulmonary function tests, can be performed in infants to assess thoracic hypoplasia. Therefore, we should have been alarmed by the constant need for a high-flow nasal cannula with a FiO_2_ of 0.4 and the presence of retractive breathing. However, we were focused on correcting the airway because of the large skull size and underestimated thoracic hypoplasia. In patients with low thoracic compliance due to conditions such as thoracic hypoplasia, additional factors impeding airway patency can easily result in mask ventilation failure. In reports on OI type II, most patients had large skulls due to hydrocephalus and thoracic hypoplasia [2,5,6]. Therefore, an anesthesia plan for OI type II should be developed considering these precautions, favoring a safe method. We suggest attempting to secure the airway with spontaneous breathing while remaining under mild sedation to avoid iatrogenic fractures due to body movements. Laryngospasm or bronchospasm are also possible factors contributing to mask ventilation difficulty. However, we consider laryngospasm unlikely when a muscle relaxant is administered. As no wheezing or upsloping capnograph was observed, bronchospasm was not considered.

Iatrogenic fractures are also a problem in the anesthetic management of OI and require attention during airway management and surgical positioning. Iatrogenic fractures occurred in only two of the total 637 surgeries reported in the studies by Rothschild et al. [1], Bojanić et al. [3], and Liang et al. [4], and no fractures associated with airway management were reported. Similarly, these were not observed in individual OI type II cases [5,6] and in our case. Therefore, bone fragility may not be a concern to the extent that overly gentle airway management is necessary.

## Conclusions

For patients with OI type II scheduled for general anesthesia, bone fragility may not be concerning to the extent that airway management becomes compromised. While attention is directed towards the management of bone fragility and large skull sizes that may affect the airway, thoracic hypoplasia should also be of concern. In addition, if patients present with poor oxygenation due to thoracic hypoplasia, the possibility of difficulty with mask ventilation due to low compliance should be considered in airway management.
